# Clinical and Laboratory Approaches to Hemophilia A

**Published:** 2015-05

**Authors:** Hassan Mansouritorghabeh

**Affiliations:** Allergy Research Center, School of Medicine, Mashhad University of Medical Sciences, Mashhad, Iran

**Keywords:** Hemophilia A, Factor VIII, Hemorrhage, Coagulation factor VIII

## Abstract

Hemophilia A is a worldwide disorder of coagulation system. It is a male disorder, yet females with hemophilia are rarely seen in communities with high rate of consanguineous marriages. The abnormalities in factor VIII gene transfer as an X-linked pattern in the family, affects as many as one-third of patients who had no family history of abnormality and thus the occurrence of a sporadic mutation could be documented. Hemorrhagic symptoms usually correlate with the plasma level of factor VIII and comprise a wide range of hemorrhagic pictures, including from fatal spontaneous bleeding in the brain to ecchymosis of the skin. The coagulation study needs to differentiate between the two types of hemophilia A and B as well as the categorization of the disease severity. In the developing countries, due to limitations in diagnostic hemostasis facilities and a scant number of experts in the field, it is estimated that noticeable numbers of undiagnosed patients with hemophilia A exist. Occasionally, we encounter undiagnosed cases by general physicians while having hemorrhagic symptoms. The purpose of this review is to recap clinical and diagnostic parameters, pitfalls, and interpretation of coagulation assay in hemophilia A.

A literature review was done in PubMed and Scopus medical search engines using the keywords “Hemophilia” and “Haemophilia”. A time limitation for the publication beyond 1995 and publication in the English language were considered. A total of 94 original articles and chapters of books was selected for the current review. Additionally, a comprehensive and up-to-date information on the clinical and laboratory features for the diagnosis of hemophilia is also presented.

## Introduction

Hereditary deficiency of each coagulation factor in hemostatic cascade is explained. Hemophilia A (HA) is the most common coagulation factor deficiency around the world with high treatment cost.^1^ It affects all ethnic populations^2^ and its prevalence varies among different countries, but estimated at a rate of 3-20 cases per 100,000 population.^3-5^ The World Federation of Hemophilia (WFH) has estimated the total number of hemophilia at about 500,000 universally, while one-third are diagnosed.^6^ About 30-33% of the new diagnosed cases of the current X-linked disorder show no family history; reflecting on its occurrence by spontaneous or sporadic mutations.^1^^,^^7^^,^^8^ Genetically, it stems from the deletion of the whole or part of gene, point mutations gene or in regulators of the gene,^9^ and major inversion of the tip of X-chromosome that is responsible for 50% of the severe form.^10^ Such variety of abnormalities in factor VIII gene result in the absence of impaired molecule production of factor VIII, which has no function or reduction of factor VIII in the plasma of the affected individuals and cause a tendency to bleed with various severities that correlate with the plasma level of factor VIII. HA or classical hemophilia accounts for about 80% of all hemophiliacs. This lifelong disorder has three phenotypes (severe, moderate and mild) that correlates with factor VIII (FVIII) levels in plasma (<1%, 1-5%, 5-30% respectively) and its clinical phenotypes.^11^^,^^12^ The lack or severe decrease of FVIII can cause joint and muscle bleeding, which is regarded as the hallmark of severe HA.^13^ Also, the production of thrombin-activable fibrinolysis inhibitor (TAFI) is reduced in HA that leads to ease distillation of fibrin clot, failing to achieve robust hemostasis, and easy rebleeding.^14^ Nowadays, with the improvement of therapeutic facilities, the median life expectancy has been increased noticeably.^15^

The purpose of the current review was to collect and summarize publications to present the state-of-the-art on clinical manifestations of hemophilia A and laboratory approaches in diagnosing the undiagnosed individuals with bleeding and suspect to hemophilia A. 

## Clinical Manifestations

The classification of HA provides a guidance to possible types of bleeding and the rate of occurrence of hemorrhagic episodes. The patients with severe form, experience spontaneous bleeding and hemorrhage after minor trauma about 1-6 times in a month, including hemarthrosis and intramuscular hemorrhage. In moderate form, the affected patients usually experience bleeding after mild to moderate injuries, while HA patients with mild form may not be diagnosed for years and bleed after surgery or major trauma.

About 70% of children who have a positive history of HA in the family are diagnosed at birth or after the first bleeding episode. At the same time, children who have a negative family history are diagnosed in situations such as post circumcision bleeding, post injection of a drug or vaccine, experience easy bruising after minor trauma, ecchymosis in buttocks at the beginning of crawling or walking, post trauma bleeding or even bleeding at the time of torn frenulum.^13^

## Hemarthrosis

Hemarthrosis is a common finding in hemophilia and is the hallmark of a severe form. Some of the patients with HA are alert at the beginning of hemarthrosis by sensing aura symptoms that consist of a warmth and tingling sensation and movement restriction of the joint. This is the best time for the injection of factor VIII concentrate to prevent bleeding. Inflation of the joint capsule, secondary to hematoma is an important cause of pain.^16^^,^^17^ The precipitation of iron in synovial of the joint and its subsequent intake by macrophages, causes destruction of cartilage and shaping jagged and rigid form to it.^18^ Recurrent intra-articular bleeding leads to synovitis and cause impaired cycle.^19^ The clinical features comprise of pain, inflammation, inflation, elevation of organ temperature, and motion restriction. They are mostly due to the production of kinins and cytokines by the leaked blood. In certain cases, arthrocentesis under sufficient replacement therapy may be objected to prevent damage to cartilage and debilitating dysfunction and pain chronically.^20^^,^^21^ Non-invasive methods such as MRI and ultrasonography may be useful in the detection of early cartilage damage.^22-24^ The frequency of hemarthrosis is completely related to the severity of HA. The patients with severe HA are usually affected by acute hemarthrosis in adulthood, which is usually due to high physical activities. Its frequency is reduced after puberty and is less observed in middle age. Despite this fact, the process of progressive destruction of joint exists in middle age.^19^ The most common joints for bleeding are the knee, elbow, ankle, and wrist. The hip joint is less affected, which might be due to its protection by massive and bulky mass of muscles. Surprisingly, small joints of the hands and vertebra are rarely affected.^25^

## Hematoma

Bleeding in muscle includes 10-25% of hemorrhagic episodes in severe HA.^13^ Muscle hematoma is regarded as the major cause of disability in hemophilia. It usually originates from trauma, but occurs spontaneously or even by post emotional stress. About 75% of the patients with severe HA experience this during their lifetime. The main manifestations of hematoma is pain and inflation, and its severity correlates with the severity of hemophilia, size of hematoma, involvement of fascia and muscle type.^26^^,^^27^ After muscle hematoma, a rapid and protective muscle spasm occurs, which is usually associated with the restriction of joint movement and pain. In addition, in traumatic hematoma, there might be a superficial ecchymosis and sore.^28^ The necrosis of muscle can be confirmed by the detection of keratin kinas, lactate dehydrogenase, amino-transferees and aldolase. The performance of ultra-sonography, computed tomography (CT) or magnetic resonance imaging (MRI) may help to determine the extent of hematoma. There is inflammation at the site of hematoma due to the proliferation of poly-nuclear cells and subsequently by mononuclear cells. The intramuscular injection can cause hematoma, thus its performance in hemophilia is subjected to sufficient replacement therapy.^13^

Iliopsoas hematoma is a life-threatening hemorrhage that may lead to death. It may be seen occasionally in the cases that are not on prophylaxis regimen.^29^ One of the most common features of iliopsoas is femoral nerve compression.^13^^,^^30^ It is usually associated with the vague symptoms of lower abdominal and upper thigh. Although the external and internal rotation of the hip is normal, but the patient is unable to extend the hip and has a distinctive gait. On examination, the hip is inwardly rotated and flexed. Iliopsoas hemorrhage may be life threatening if it is associated with the high volume of blood loss in the retroperitoneal space.^31^

## Central Nervous System (CNS) Bleeding

The bleeding in CNS is a serious cause of morbidity and mortality in hemophilia. The affected patients under 18 years old may encounter more complications.^32^^,^^33^

The signs such as shock, hypotension, lethargy, and anemia may present as an unspecific manifestation of bleeding, while severe sign and symptoms include painful headaches or neck pain, repeated vomiting, sudden problem in walking or arm movement, sleepiness and behavioral changes, seizures or convulsions and double vision.^34^

Before introducing new therapeutic options, about 75% of intracranial hemorrhage caused death; however, nowadays this is reduced to about 30%. It is more frequent in children and young adults, which is to do with more physical activities and higher possibility of direct trauma to the head. The performance of CT or MRI may detect multiple unpredicted bleeding sites.^13^ Prompt diagnosis and early commence of treatment can lessen mortality and prevent neurological sequelae.^32^

## Gastro-Intestinal Bleeding (GIB)

GIB occasionally occurs in hemophilia. It may occur spontaneously or secondary to common causes of GIB. After managing a GIB episode, a work up is needed to detect causative cause.^13^ Hemarthrosis and pain in patients with HA are associated with the use of anti-pain medications that may ameliorate the risk of GIB. It is estimated that the risk of upper GIB is 5 to 10 times higher in normal population not taking anti-pain drugs. In a report that evaluated the causes of death in the United Kingdom, upper GIB was the cause of 13 deaths among 1,190 deaths.^35^^,^^36^ Helicobcter pylori infection can increase the frequency of GIB in patients with hemophilia.^37^

## Hematuria

Hematuria is a troublesome burden in patients with hemophilia. A flank and sudden pain in the abdomen of a HA patient must be investigated regarding the possibility of hydronephrosis or ureteral obstruction secondary to hemorrhage.^13^ The renal disease is considered as a rare complication in HA,^38^ despite this, there is a discrepancy in the reported frequency of hematuria in hemophilia (2.9%) as described by Kulkarni et al.^38^^,^^39^

## Post Circumcision and Tooth Extraction Bleeding

Circumcision practice and dental extraction are the two invasive procedures that challenge the hemostasis system. Post circumcision bleeding is common among babies with hemophilia who do not receive a proper amount of coagulation factor prior and post-practice. It may be the first bleeding manifestation of a baby with a mild hemophilia. Not only in neonate or babies with a negative past history of hemophilia, but also in babies with positive family history, circumcision practice must be delayed until the primary screening test results are known. Note that, 30% of patients with HA are sporadic cases.^13^^,^^40^ Also, hemorrhage may still occur despite adequate replacement therapy in circumcision practice and the circumcised patient should be observed in the medical center to allow prompt intervention in case of bleeding.^41^

The risk of post tooth extraction bleeding is great and can be life threatening.^42^ Oral cavity has fibrinolysis activity that may exacerbate bleeding. Dental extractions need treatment with coagulation factor and an antifibrinolytic agent.^43^

## Other Bleeding Episodes

Bleeding in vertebra,^44^ gums,^42^ and sublingual hematoma^45^ may be rarely observed in HA.

## Reduced Bone Density

There are many reports on the status of bone density in HA that show severe, moderate and mild forms of HA may occur with osteopenia and osteoporosis. The osteopenia and osteoporosis do not usually have any obvious signs until they lead to a sudden fracture of the affected bone. One of the main reasons for the reduced bone density in HA may be reduced physical activities secondary to joint problems.^46-48^

## Vaccination in Hemophilia

In our region, we routinely encounter HA patients with a history of infection with hepatitis B virus (HBV) and hepatitis C virus (HCV).^5^^,^^13^ In recent decade, considering proper screening and selection of blood donors as well as various recruited tests on plasmas, transition risk of HBV, HCV and human immunodeficiency viruses (HIV) has been largely reduced.^49^In the developed countries, where there is a better budgetary system for health issues, hepatitis A virus (HAV) is considered as a risk factor both in donor selection phase and also the process of blood products and preparation.^50^

There is a concern about the development of inhibitor after vaccination in young HA patients during 50-100 days after the infusion of coagulation concentrates.^51^ Overall, based on the source of coagulation concentrates and available data on the transmission of the virus by plasma-derived products, two consideration points are recommended regarding vaccination in HA patients:

- The HA patients who regularly and merely infuse recombinant concentrate; there is no need to be vaccinated against HBV and HAV. Although due to the existence of other sources of acquiring HBV and HAV, they should consider this at a later stage of life.
- The HA patients who regularly infuse plasma derived concentrate would be vaccinated against HAV and HBV using the established schedule.^52^

## HA and Females

**Figure 1:**
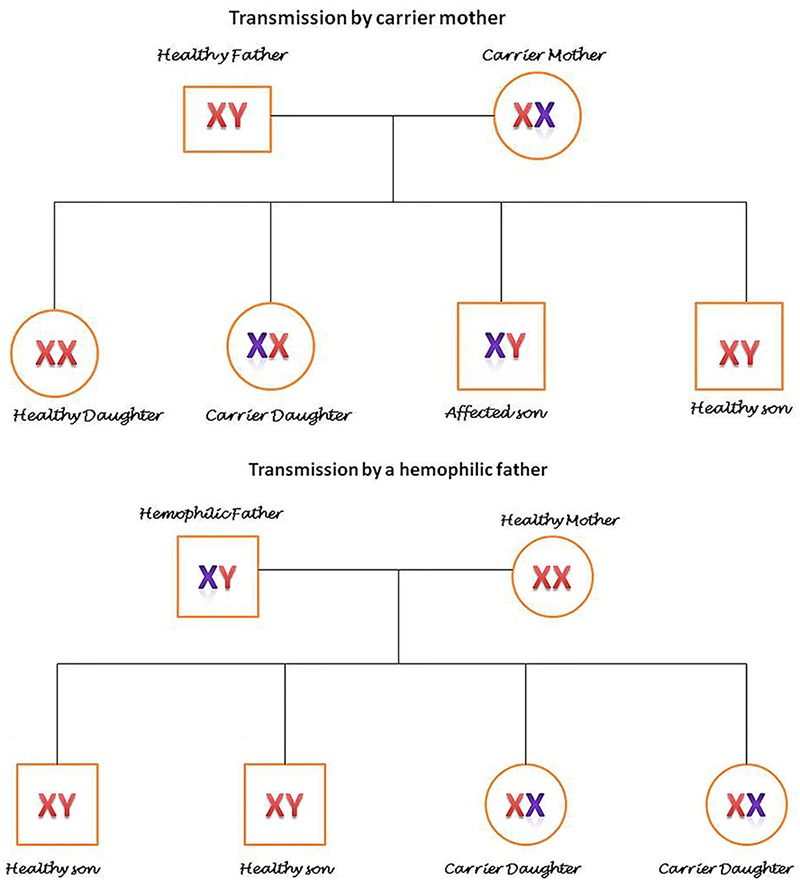
The schematic patterns of hemophilic gene that show how it transfers from carrier mother and affected father.

**Figure 2:**
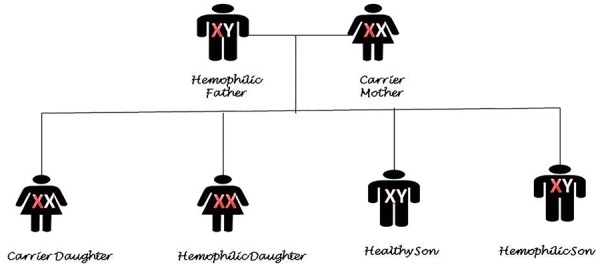
The schematic pattern of affected genes from a carrier mother and a hemophiliac father that have been delivered to offspring.

Hemophilia is an X-linked disorder and mainly affect males, whereas females are mainly carriers^53^ (figure 1). However, in certain conditions, it can affect females too: (i) X-chromosome lionization,^54^^,^^55^ (ii) inactivation of normal X chromosome in a carrier, (iii) Turner’s syndrome^56^^,^^57^ (iv) offspring of a carrier mother and father with hemophilia (figure 2).

It is reported that about 617 hemophiliac female existed in the USA during 2007. The female-to-male ratio estimated at 1:32.^15^ Awareness of the carrier status may be useful in protecting against neonatal head bleeding due to the assisted delivery using forceps or vacuum.^58^ Using genetic consulting and prenatal diagnosis test, it is possible to have healthy male delivery.^59^

## Ordering Screening Tests

Clinical pictures in HA, HB and many other hemorrhagic disorders are similar.^60^ It seems hard to make a diagnosis of the underlying cause of bleeding in new cases without the assistance and the availability of hemostasis tests. The only possibility is to gather exact family history and find a diagnosed case with confirmed diagnosis among the family members. Nevertheless, verification of hemostatic tests will be needed. Choosing an appropriate screening test should be based on reviewing the type of current and past bleeding episodes, history of bleeding in the family and demographic results (including sex, age at first time of hemorrhagic episode), and consanguineous marriage status of the parents.^13^

The screening tests, including complete blood cell count, activated partial thromboplastin time (APTT) or partial thromboplastin time (PTT), thrombin time (TT), prothrombin time (PT) and vWF:Ag tests are rational for the evaluation of hemostasis. The prolonged APTT or PTT with normal PT and bleeding time are typical findings.^61^ A prolonged screening test needs to be followed by studying coagulation factors to establish not only the net diagnosis, but also to determine the severity of the disorder and monitoring replacement therapy.^62^

## Key Points in Blood Venipuncture

A plasma sample derived from clean venipuncture without trauma, air bubbles and contamination with tissue fluids is necessary. The sample should be free of visible hemolysis otherwise; it should be discarded without studying.^63^ Improper samples, such as sampling by indwelling catheter, result in the contamination of the sample by anticoagulant and infusible fluids. This may lead to false results and eventual biased diagnosis.

The obtained whole blood is mixed with citrated sodium salt (3.2-3.8%) with a ratio of 1:9 volume/volume. The citrate salts can work as calcium chelators to absorb it. Consequently, by removing calcium from the coagulation cascade, the blood cannot clot. Indeed the ratio of citrate is in accordance with the plasma volume, thus, it is important that the amount of citrate to be adjusted for anemic and polycythemic patients^64^ according to following formula:

Amount of citrate (ml) needed for 1 ml blood=(100-hematocrit)**/**(595-hematocrit)

The blood volume in neonates is small and even little volume of blood sampling may lead to noticeable consequences. Therefore, ordering hemostatic tests must be logical and sampling should be done in special pediatrics tubes with lower volume of citrate and thus the lower volume of blood.^65^

The sample should be rotated completely to ensure immediate sufficient mixture with citrate. The best option is to collect blood in plastic tubes or siliconized borosilicate glass containers. The sample must be centrifuged for 15 minutes at 1500×g. The sample should be tested as soon as possible due to the decrease in labile factors V and VIII; otherwise, it can be refrigerated for up to 4 hours, for longer time it should be frozen.^61^

## The PTT Assay

The PTT assay is requested for three major goals; (i) detection of inherited or acquired coagulation factor(s) deficiency in intrinsic pathway, (ii) monitoring of heparin therapy, and (iii) detection of lupus anticoagulant. It is affected by a decrease in plasma level of the coagulation factor(s) involved in intrinsic (XII, XI, IX, VIII, high molecular weight kininogen, and prekallikrein) and common (I, II, V and X) pathways. The secondary hemostasis is necessary to consolidate platelet plug that has been built in primary hemostasis by cross-linked fibrin.^66^

In PTT procedure, patient’s plasma is incubated with a PTT reagent (that is a surface activating agent such as clay, kaolin, silica, ellagic acid, or crude phospholipid) for about 3 minutes at 37ºC. During pre-incubation time, contact factors (XII and XI) are activated in the presence of high molecular weight kininogen and prekallikrein. The activated factor XI, converts factor IX to IX_a_. Then pre-warmed 0.02 molar calcium chloride is added to the mixture that results in the activation of factor X with the help of IX_a_/VIIIa. Subsequently, X_a_/V activates prothrombin (II) to thrombin (IIa) is followed by conversion of fibrinogen (I) to fibrin (I_a_), which is the endpoint of PTT assay. The fibrin monomers polymerizes to fibrin strands and confirmed by the effects of factor XIII [(XII, Kallikerin, XI)→IX_a_+VIII_a_→Xa+V_a_→II_a_→I_a_→Clot].^67^

It is recommended to run a normal and abnormal plasma samples for quality control at the beginning and end of the test or with each batch of test. This is also obligatory in the case of changing reagents and instruments. Avoid freezing and thawing samples, as this may lead to erroneous results by releasing phospholipid from cell membrane of platelets.^68^

Each hemostasis laboratory must establish a normal reference value by using instruments, the method of sampling and the testing technique used by the laboratory.^69^

In selecting APTT reagent, the following two items should be considered: (i) the sensitivity of the kit to detect the severity of hemophilia and (ii) the capability of the reagent to measure plasma levels of a coagulation factor.^70^

## Interpretation of PTT

The most PTT reagents may not detect mild deficiency of the coagulation factor (30-50% of normal), while they are sensitive to deficient levels (10-20%). The usual PTT reagents are more sensitive to deficiency of factors VIII, XI, and XII rather than factor IX. When selecting PTT reagent, it is important to consider the main goal of usage. For example, in hemostasis laboratory at hospitals where many patients with bleeding disorders may need a PTT reagent, it is critical to detect factor deficiency. However, in gynecological hospitals, large numbers of obstetrical patients may need a PTT that is more sensitive to lupus coagulants. The international sensitivity index (ISI) determines the amount of reagent sensitivity and the kit to detect hemostatic abnormalities. Lower ISI usually indicates the more sensitive the reagent. As a criterion, ISI of 1.8 to 2.4 has low sensitivity, ISI of 1.4 to 1.8 has median sensitivity, and ISI 1.0 to 1.4 has high sensitivity. The World Health Organization (WHO) suggests using a kit with ISI<1.5.

When a PTT result is prolonged, the sample must be evaluated for turbidity, icteric, lipemic or hemolyzed visage. Such specimen may lead to erroneous results. In addition, factor VIII is one of the C-reactive proteins and its high level may mask co-existing mild deficiency status of other coagulation factor in the intrinsic pathway. The factor VIII level increases after excitement, exercise, and epinephrine injection.^13^

In a situation of activated sample (poor quality blood sampling), chronic disseminated intravascular coagulation, and increased plasma levels of factor VIII, the PTT may be shortened.^71^

Physiologically, neonates have reduced levels of the majority of procoagulant and anticoagulant factors, except for factor VIII. This leads the hemostatic system to be simply overwhelmed.

Interpreting abnormal hemostatic tests, one should note that neonates might be affected by the state of maternal health and the medications used by the mother during labor.

In acute inflammatory reaction, there is an elevated level of fibrinogen that can shorten PTT result. Also, consider that oral contraceptives, estrogen, pregnancy, heparin, coumarin type drugs, naloxone, and asparaginase can act as interfering substances and influence PTT results. 

## Mixing Study

It is important to perform a mixing study for the samples showing prolonged PTT, to determine the cause. The mixing text is done by mixing 1:1 volume/volume ratio of patient’s plasma with the normal pooled plasma. After mixing, the PTT is repeated and the result should shorten close to the reference value (at least to less than 50% of the clotting time), accordingly it shows the deficiency of the clotting factor. If it does not return to the reference limits, further evaluation for heparin or inhibitor is needed.^62^

The inhibitors are neutralizing antibodies, mainly from IgG class, that can bind to factor VIII in the plasma of HA patients or healthy individuals and cause impaired function of factor VIII in circulation. They can be measured by antigen-antibody methods (enzyme-linked Immunosorbent assay, ELISA) or by functional evaluation, coagulation based methods (Nijmegen or Bethesda assays). They are expressed as a Bethesda unit (BU) where one BU shows the amount of factor VIII inhibitor that can neutralize 50% of the activity of factor VIII in plasma after 2 hours incubation at 37ºC.^72^

In emergencies, where there is unexpected bleeding, mixing study may be useful to identify the presence of heparin. Nevertheless, corrections of PTT doses not rule out the existence of heparin in plasma. In such situation, heparin assay should be done or plasma sample should be treated with heparinase and then the test should be repeated.^73^

If mixing tests fail to return to the normal reference and APTT is still prolonged, the existence of inhibitor is suspected. For factor deficiency situation, an assay of the suspected factor(s) should be carried out.^74^ Test for the detection of lupus anticoagulant and screening for inhibitor must be done in a later stage. Factor VIII inhibitor may be time dependent and in mixing procedure, two hours incubation is needed. Also, lupus like anticoagulants are relatively weak and a mixture of 25:75 of normal and test plasma are needed to reveal them.^62^^,^^72^^,^^75^

## Factor VIII: C

It is a single chain protein with 2332 amino acid and 280 KD weight,^76^^,^^77^ which is synthesized mainly by the liver and spleen. The relevant gene is located in the long arm of X chromosome (Xq26).^14^ It forms a complex with von Willebrand factor (vWF) with a ratio of 1:50-100 in circulation. The vWF molecule has two roles, as a transporter and a stabilizer for FVIII. In the presence of vWF, half-life of FVIII is about 12 hours,^78^ while without it, the half-life decreases to about 2 hours.^79^

FVIII has 3 A domains and 2 C domains that connect together using the B domain. The secreted FVIII present as two-chain heterodimer as a result of cleavage at B-A3 junction site. The B chain is highly glycosylated and includes 18 of the 25 potential N-linked glycosylation sites.^80^ It is activated by cleavaging at Arg in positions 372, 740 and 1689 by thrombin (FIIa). The activated FVIII including A1/A3-C1-C2 that is connected to the A2 domain via weak ionic interaction.^81^

FVIII is the largest and most unstable coagulation factor with the capability to adsorb onto surfaces of foreign materials. It degrades with fluctuations of pH, ambient temperature, and exposure to proteolytic enzymes of plasma.^70^

## Factor VIII Assay

Factor VIII assay is necessary for the diagnosis of HA, determination of phenotype, monitoring FVIII treatment and accurately finding out concentrate potency.^75^^,^^77^ In patients with mild HA and new diagnosed cases, especially if there is no family history, the level of vWF:Ag must be detected. The abnormal vWF may bind to FVIII and cause more rapid catabolism of FVIII in circulation.^76^^,^^82^ Nowadays, three major methods are used for the measurement of factor VIII, namely one-stage methods, classical two-stage assay and chromogenic assays.^81^

While clinical and hemostasis laboratories usually use one-stage method due to its simple procedure and calibration of instruments, FVIII manufacturers use two-stage method, since there is no need for deficient plasma and due to its accuracy.^81^^,^^83^ Also, the chromogenic method is the reference by the International Society of Thrombosis and Hemostasis (ISTH) and the European Pharmacopoeia.^82^ Chromogenic assays need sufficient activation of FVIII and the presence of factor IXa, calcium, and phospholipids.^84^ Usage of platelet rich plasma in one-sage methods can correspond with the chromogenic assay.^85^^,^^86^

The FVIII assay by different methods, particularly in severe type, gives similar results. However, there are noticeable reports of discrepancy among more than 30% of FVIII assays in the mild and moderate HA with distinct subclasses of missense mutations.^87-90^ The FVIII level may be increased due to inflammation and stress, therefore, repeated assay may be needed to establish the diagnosis. The performance of the internal and external quality control procedures can be very useful.^91^^,^^70^

## Further Tests in HA

In mild and moderate HA, reduced FVIII secondary to vWD should be excluded. In such cases, taking family history to reveal a recessive inheritance type and assays for vWF:Ag and ristocetin cofactor (RiCof) should be done simultaneously.^92^^,^^93^ In vWD type 2N low levels of factor VIII, vWF:Ag and RiCof may be observed.^94^

Beside hemostatic tests, detection of possible transmission of blood borne viruses and the evaluation of overwhelming symptoms related to them, including the liver function tests must be considered. 

## Conclusion

Hemophilia is a bleeding disorder with global distribution. It affects mainly men and rarely seen in women. The bleeding manifestation varies from lethal hemorrhagic episodes in CNS to superficial ecchymosis. Performing a complete physical exam, gathering entire past medical history of the suspected cases and the family, and collecting demographic data are important in diagnosis. It should be followed by appropriate hemostasis assays to confirm diagnosis. Hemostasis laboratories should pay sufficient attention to the characteristics of the screening kits that they deploy for the evaluation of patients to cover the spectrum of the suspected cases with hemophilia and avid missing mild cases. 
